# Application effect of gastrointestinal bundle nursing on the protection of gastrointestinal function in patients with gastric cancer

**DOI:** 10.1097/MD.0000000000034308

**Published:** 2023-07-21

**Authors:** Xiao-Ning Wei, Wen-Yan Cai, Kai-Ling Wu, Fei-Ge Zeng

**Affiliations:** a Department of Gastrointestinal Oncology Surgery, The First Affiliated Hospital of Hainan Medical University, Haikou, Hainan, China; b Department of Urology Surgery, the first affiliated hospital of Hainan Medical University, Haikou, Hainan, China.

**Keywords:** emotional response, enhanced recovery after surgery: bundled care, gastric cancer, gastrointestinal function, stress response

## Abstract

Evidence-based nursing practice was used to formulate the enhanced recovery surgery bundle nursing strategy and apply it to patients with gastric cancer, to explore its safety, effectiveness and feasibility in perioperative gastrointestinal function protection in patients with gastric cancer. Selected the clinical medical records of 100 gastric cancer patients treated in our hospital from June 2019 to June 2021 as the research objects, and divided them into the control group and the observation group with 50 cases in each group according to the random number table. Among them, the control group was given routine nursing measures for nursing intervention, and the observation group was given gastrointestinal enhanced recovery surgery cluster nursing on the basis of the control group. The differences in stress response, gastrointestinal function protection, negative emotions and pain scores of gastric cancer patients before and after nursing were compared between the 2 groups. The postoperative bowel sounds recovery time, first anal exhaust, and first defecation time in the observation group were lower than those in the control group, and the differences were statistically significant (*P* < .05). Before nursing, there was no significant difference in the scores of stress response changes between the 2 groups (*P* > .05). After nursing, heart rate (HR), mean arterial pressure (MAP), norepinephrine (NE), and epinephrine (E_2_) in the observation group were lower than those in the control group, and the difference was statistically significant (*P* < .05). The pain scores of the 2 groups were significantly improved at different time points, and the observation group was significantly less than the control group, and the difference was statistically significant (*P* < .05). Gastrointestinal enhanced recovery surgery bundle nursing can effectively improve the gastrointestinal function of patients with gastric cancer, improve the emotional response and stress response of patients, and has certain reference value for the nursing of patients with gastric cancer.

## 1. Introduction

Enhanced recovery after surgery (ERAS) refers to the promotion of postoperative recovery of patients through a series of perioperative management modalities, and postoperative promotion of gastrointestinal function recovery and early transoral feeding is one of the important measures of ERAS.^[1]^ Laparoscopic radical gastric cancer surgery is less invasive and has rapid postoperative recovery, which is in line with the ERAS concept.^[2]^ However, intraoperative vagal hyperfunction, hypotension, surgical straining, and postoperative medication can cause gastrointestinal dysfunction, often leading to reactions such as delayed digestion and exhaustion, nausea and vomiting.^[3]^ Gastric cancer is a malignant tumor with a high incidence rate and a high mortality rate. Gastric cancer is not easily detected at the early stage of development, and the diagnosis rate is low, and once detected, the disease has reached the middle and late stages. Most clinical treatments for malignant tumors are based on surgery and chemotherapy, which is a highly stimulating treatment, and the toxic side effects of antitumor drugs can lead to gastrointestinal reactions after treatment, further causing complications such as difficulty in food intake and poor immunity.^[4]^ Therefore, postoperative nursing interventions for gastric cancer patients play a key role in ensuring their smooth recovery.^[5]^ Studies have found that conventional nursing interventions are poorly targeted and can no longer meet the nursing needs of gastric cancer patients.^[6]^

How to promote the recovery of gastrointestinal function and strive for early enteral nutrition is the key to the rapid recovery of gastric cancer patients.^[7]^ Clustered interventions are a structural approach to improve the quality of care and improve patient outcomes.^[8]^ While implementing conventional nursing approaches, it can also pool strengths and complement each other to improve the effectiveness of care and improve patient prognosis.^[9]^ Therefore, we studied the use of evidence-based nursing practice to develop an accelerated rehabilitation surgical cluster nursing strategy and apply it to gastric cancer patients to explore its safety, effectiveness and feasibility in the perioperative gastrointestinal function protection of gastric cancer patients and to provide a certain reference basis for the clinical care of gastric cancer patients.

## 2. Materials and methods

### 2.1. Research objective

The clinical records of 100 cases of gastric cancer patients admitted to our hospital from June 2019 to June 2021 were selected as the study subjects and divided into 50 cases each of control group and observation group according to the random number table. The selected gastric cancer patients all met the diagnostic criteria of gastric cancer as described in the Chinese Expert Consensus on Difficulties in the Diagnosis and Treatment of Gastric Cancer, and the properties and characteristics closely related to the nature and biobehavioral characteristics of gastric cancer itself, such as the lesion being cancer, the degree of differentiation of the tumor and the expression of specific molecules, were clarified through gastroscopy for lesion site biopsy and pathological examination.^[10]^ This study was approved by the Medical Ethics Council of The First Affiliated Hospital of Hainan Medical University.

### 2.2. Inclusion and exclusion criteria

Inclusion criteria: patients aged 30 to 80 years old, diagnosed with gastric cancer by medical history, clinical symptoms and preoperative gastroscopic biopsy; patients with normal consciousness, normal language ability, normal mental status and normal cognitive function; patients without contraindications to surgery, with elementary school education or above, able to live normally and with good compliance. Exclusion criteria: patients who received emergency surgical treatment for acute complications caused by gastric cancer, such as gastric bleeding and gastric perforation; patients who received palliative surgery, patients who were referred for open surgery; patients with mental illness, consciousness disorders and communication disorders, and patients with combined serious heart, liver, kidney and other systemic diseases.

### 2.3. Intervention methods

#### 2.3.1. Clustered nursing interventions.

Among them, the control group implemented the routine nursing intervention of accelerated rehabilitation surgery, namely early postoperative care: after the surgery, the patient is still in an unconscious state due to anesthesia, at this time, nursing staff can massage the patient calves to promote blood circulation; after the patient is awake, the patient is instructed to appropriately flex and extend the limbs, and 1 day after surgery, the amplitude of limb activities can be increased, such as lifting the arms, stirrups, leg lifts, etc, and the activity interval is 2 hours once, 5 minutes/time. If the patient recovers well, he can get out of bed in 2 days after surgery. The nursing staff should give assistance and guidance to the patient when he gets out of bed for the first time, first instruct the patient to sit quietly at the bedside, and then decide whether he can get out of bed with his own condition. Simulated feeding training: patients were given sugar-free chewing gum for simulated feeding training 10 hours after surgery, 3 times/d, 2 capsules each time, about 10 minutes per training, those who could not chew gum could use cooked tea instead. Postoperative abdominal massage: the patient was given abdominal massage after 6 hours of anesthesia wakefulness, the patient took a supine position, bending knees, nursing staff 4 fingers together, avoiding the incision, from the umbilicus to perform clockwise massage, and then repeatedly massage from both sides of the incision up and down after completion, 3 times/day, 10 minutes each time; massage while actively communicating with the patient, according to the patient feelings from light to heavy operation. Foot care: instruct the patient to do foot bath before bedtime, the water temperature is controlled at about 50°C, the water level is not over the feet, soak for 10 minutes and then scrub, each foot bath for about 20 minutes, dry and pay attention to the foot warmth. Prevention of hypercapnia: assist patients to turn regularly after awakening from surgery, generally once every 2 to 4 hours, repeatedly from right-sided position, flat position and left-sided position in sequence, meanwhile, instruct patients to breathe deeply and expel CO2.

#### 2.3.2. Gastrointestinal accelerated rehabilitation surgical cluster care.

The observation group implemented gastrointestinal accelerated rehabilitation surgical intensive care on the basis of the control group, namely early activities: before the anesthesia was awake, help the patient to massage the calf; 6 to 12 hours after surgery, instruct the patient to carry out local flexion, extension and external rotation activities of the upper and lower limbs, once every 2 hours for 5 minutes. 24 to 72 hours after surgery, gradually increase the amount of activities: the upper limbs can be gripped, pulled and lifted. The lower limbs can be lifted, stirred, kicked, etc, once every 2 hours for 5 minutes, in preparation for early bedtime activities. Patients whose condition permits can get out of bed 24 to 48 hours after surgery, first instruct the patient to sit quietly at the bedside for 5 minutes, and then assist the patient to get out of bed according to the situation. The activity time should be arranged in the morning, at noon and before going to bed at night, and the amount of activity should be based on the patient not feeling fatigue. Chewing gum to simulate eating: start chewing sugar-free gum 8 hours after surgery, once a day in the morning, noon and evening, 2 capsules/time, 5 to 10 minutes each time. those who cannot tolerate gum can chew boiled green tea leaves. abdominal massage: 6 hours postoperative abdominal massage: patients lying supine, hip flexion, knee flexion, the massager 4 fingers together, the umbilicus as the center, avoiding the incision clockwise massage, while along both sides of the incision from top to bottom, from bottom to top back and forth massage, 15 minutes/time, 3 times/day, until the patient resumed anal exhaust. The operation process pay attention to ask the patient feeling, the strength from light to heavy, to the extent that the patient tolerates, while paying attention to keep warm. Prevention of hypercapnia: After the patient wakes up from anesthesia, take a semi-recumbent position and assist the patient to turn over once in 2 to 4 hours, in the order of left lateral position, flat position and right lateral position, and instruct the patient to practice deep breathing and effective coughing to promote CO2 expulsion. Anal retraction training: Patients start anal retraction training 1 day after surgery, and train once every 2 hours according to the patient condition, 10 minutes/time. Patients are given professional dietary health education to ensure that they master the relevant dietary knowledge. Patients can be given health education through illustrated comic strips at the early stage of admission, so that they know the basic principles of diet and dietary precautions in advance and are psychologically prepared for postoperative diet; a dietary health handbook is issued after surgery to further improve patients’ mastery of dietary knowledge, and detailed answers are given to those who ask questions and patients are instructed to At the same time, the patients’ diets were monitored, such as observing the types and amounts of their diets per day and per meal, and randomly asking relevant questions, and correcting patients immediately if they answered incorrectly.

### 2.4. Observation indicators

Stress response: Three time points were selected to monitor the stress response indexes, including heart rate (HR), mean arterial pressure (MAP), and plasma norepinephrine (NE) and epinephrine (E_2_) by high performance liquid chromatography at 1 day before, during, and at the end of gastric cancer. The higher the score, the more severe the pain. These indicators were measured at 2, 6, 12, and 24 hours after surgery. The Cronbach *α* values measured before the use of the above scales were all >0.914. Patients with gastric cancer filled out the scales independently under actual conditions not influenced by any internal or external factors.

### 2.5. Statistical analysis

All data from our study were checked using Excel double entry, and SPSS 23.0 was used for statistical analysis, setting the test level *α* = 0.05 and considering *P* < .05 as a statistically significant difference. Statistical descriptions of measurement data obeyed normal distribution were described by mean ± standard deviation, and those not obeying normal distribution were described by median (interquartile spacing), and count data were described by frequency and composition ratio. General patient data were analyzed: categorical data were analyzed by chi-square test, continuity-corrected chi-square test, and Fisher exact probability method; measurement data were analyzed by *t* test. Obedience to normal distribution, paired samples *t* test was used for within-group comparisons and 2 independent samples *t* test for between-group comparisons; disobedience to normal distribution, nonparametric test-Wilcoxon signed-rank test was used for within-group comparisons and rank sum test was used for between-group comparisons for analysis.

## 3. Results

### 3.1. Demographic characteristics of participants

There was no significant difference in the clinical data of the 2 groups of patients, among which the clinical data such as gender, age, history of drinking, history of chronic gastritis, operation time, tumor size, tumor type, clinical stage, differentiated, and postoperative complications of the observation group were not different from those of the control group, and there was no statistical significance (all *P *> .05) (Table 1). However, the length of hospital stay in the observation group was significantly shorter than that of the control group (*P* = .002) (Table 1).

**Table 1:** Comparison of general data of 2 groups of patients.

Variables	Control group (n = 50)	Observation group (n = 50)	χ^2^*/t*	*P*
Gender (male/female)	26/24	25/25	0.04	.841
Age (yrs	66.31 ± 3.51	65.30 ± 3.52	1.437	.154
History of drinking			0.832	.362
Yes	11	15		
No	39	35		
History of chronic gastritis			1.442	.229
Yes	27	21		
No	23	29		
Operation time (d)	3.62 ± 0.58	3.59 ± 0.62	0.891	.435
Hospitalization time (d)	19.14 ± 2.99	15.23 ± 3.14	1.647	.002
Tumor size (cm)	3.92 ± 2.85	3.71 ± 2.68	1.319	.196
Type of tumor			0.897	.826
Fundus of stomach	12	13		
Cardia cancer	11	10		
Pylorus	15	13		
Gastric body	2	4		
Clinical stage			0.072	.995
I	10	11		
II	15	15		
III	21	20		
IV	4	4		
Differentiation of gastric tumor			0.094	.954
Well differentiated	7	6		
Moderately differentiated	15	15		
Poorly differentiated	28	29		
Postoperative complications				
Anastomotic leakage	1	0	1.01	.315
Postoperative bleeding	3	1	1.042	.307
Ileus	1	0	1.01	.315
Pneumonia	2	1	0.344	.557

### 3.2. Recovery of gastrointestinal function

**Figure 1.**
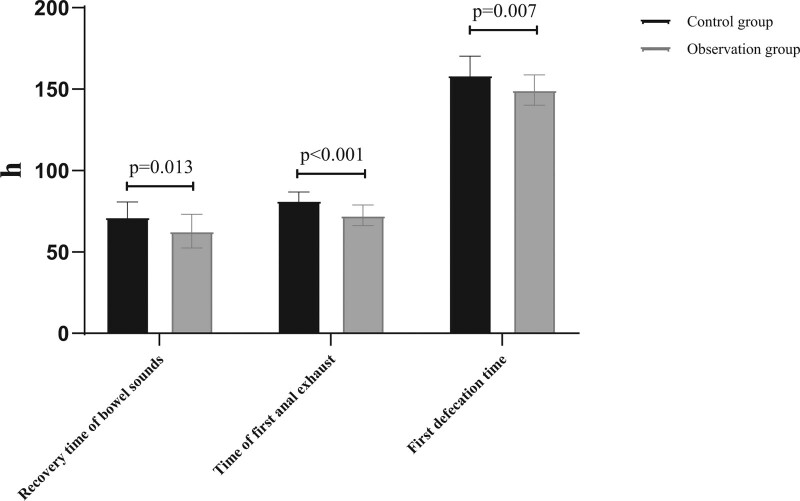
Recovery of gastrointestinal function between the 2 groups.

The postoperative bowel sounds recovery time, time of first anal exhaust, and first defecation time in the observation group were significantly lower than those in the control group (*P *< .05), as shown in Figure 1.

### 3.3. Comparison of stress response change scores

**Figure 2.**
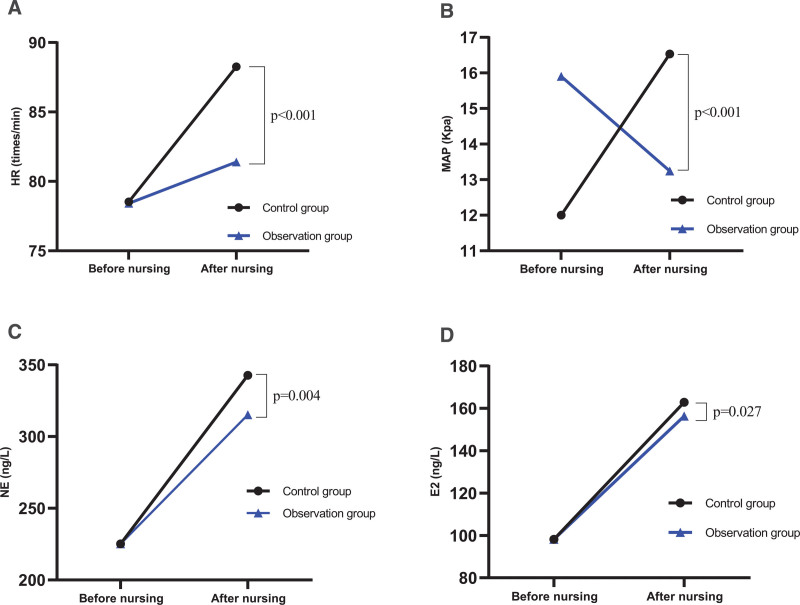
Comparison of stress response change scores between the 2 groups.

Before nursing, there was no significant difference in the scores of stress response changes between the 2 groups (*P *> .05) (Fig. 2). After nursing, HR, MAP, NE, and E2 in the observation group were significantly lower than those in the control group, as shown in Figure 2.

### 3.4. Comparison of pain scores

**Figure 3.**
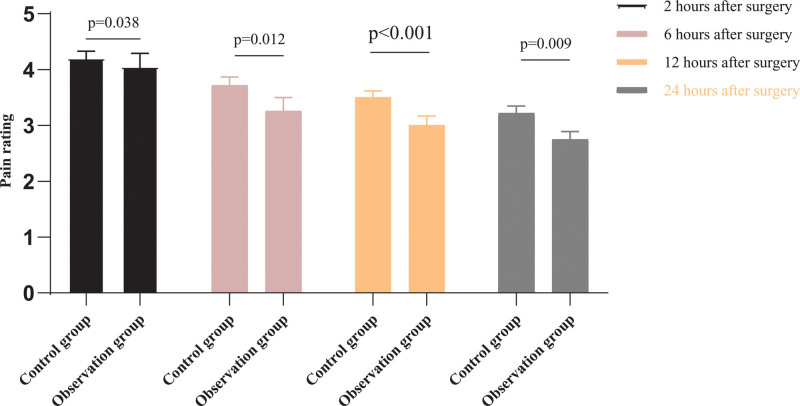
Comparison of pain scores between the 2 groups.

The pain scores of the 2 groups of patients were significantly improved at 2, 6, 12, and 24 hours after surgery, and the observation group was significantly less than the control group (*P* < .05), as shown in Figure 3.

## 4. Discussion

The speed of postoperative gastrointestinal function recovery is one of the factors affecting the postoperative hospital stay of patients undergoing gastric cancer surgery.^[11]^ Although laparoscopic radical gastric cancer surgery has advantages such as less trauma and faster recovery compared with traditional open surgery.^[12]^ However, due to the large surgical invasion of radical gastric cancer surgery, it can lead to gastrointestinal tract injury to a certain extent, causing postoperative gastrointestinal function decline and even gastrointestinal dysfunction, which is not conducive to rapid postoperative recovery.^[13]^ However, there is a lack of therapeutic methods and corresponding nursing measures to promote the recovery of intestinal function after gastrectomy, and seeking targeted nursing measures to promote the rapid recovery of postoperative gastrointestinal function in patients is the focus of nursing work.^[14]^ The reasons for this may be that early postoperative exercise helps to promote the metabolism of all body organs and effectively improve autonomic regulation.^[15]^ It also reduces the inhibitory effect of sympathetic nerves on the function of the gastrointestinal tract, increases the facilitative effect of parasympathetic nerves on the gastrointestinal tract, enhances the motor function of the gastrointestinal tract, and can effectively reduce the feeling of abdominal distension.^[16]^ Studies have shown that early bed and out-of-bed activities in postoperative patients with no contraindications to activity while awake can promote the recovery of gastrointestinal function.^[17]^ Chewing gum can mimic eating and promote gastrointestinal motility by stimulating the vagus nerve.^[18]^ Meanwhile, the intestinal paralysis of patients after gastrointestinal tumor resection is often dynamic rather than mechanical, so the gastrointestinal mucosa can absorb digestive juices normally, and chewing gum can induce the release of various gastrointestinal hormones and increase the secretion of saliva and pancreatic juice through vagus nerve stimulation, which can maintain the normal absorption function of the gastrointestinal tract.^[19]^ Patients use only masticatory muscles during chewing gum, without swallowing food, which does not lead to related complications.^[20]^ It has been suggested that chewing gum constituents (hexitol) play an important role in improving postoperative gastrointestinal paralysis.^[21]^ Massage to the abdomen accelerates blood circulation and facilitates recovery of gastrointestinal function, while promoting gastrointestinal motility and gas expulsion.^[22]^ The establishment of CO2 pneumoperitoneum during laparoscopic surgery can significantly elevate the diaphragm, compressing lung tissue and affecting pulmonary ventilation and air exchange functions, and the restricted movement of the diaphragm leads to a decrease in tidal volume and CO2 retention, causing an increase in the partial pressure of arterial blood carbon dioxide.^[23]^ Postoperative administration of semi-sitting position and regular change of position can reduce cardiopulmonary compression, and deep breathing can increase tidal volume and facilitate CO2 expulsion.^[24]^

Our study found that accelerated rehabilitation surgical cluster care of the gastrointestinal tract can effectively improve gastrointestinal function in patients with gastric cancer. The reasons for this were analyzed as follows: gastric cancer is often associated with nutritional risk and impaired gastrointestinal function, and this leads to poor clinical outcomes. Some scholars have shown that interventions with clustering nursing measures are effective in ICU patients.^[25]^ In our study, the incidence of malnutrition in patients was effectively reduced through the implementation of clustering interventions, including the establishment of a nutritional support group, calculation of nutritional intake, development of individualized nutritional protocols, and real-time assessment, which systematically provided a comprehensive assessment and prevention of each factor that may affect the patient poor nutritional status during chemotherapy.^[26]^ The implementation of clustered care measures is key to determining the effectiveness of care, and patients are systematically evaluated as a whole.^[27]^ Clusterized care is then administered and nursing staff are trained before the intervention is implemented to ensure proper implementation of care measures and to improve standardization and uniformity.^[28]^ One study applied clustered gastrointestinal functional interventions to the care of patients undergoing radiotherapy for esophageal cancer and showed that it improved the nutritional status of patients, which is consistent with our findings.^[29]^ The real-time attention of the nurse to the patient also improves the patient being valued.^[30]^ At the same time, this form of intervention allows for real-time assessment and facilitates timely adjustment of the dietary regimen according to the patient nutritional status, constructing a comprehensive circle of protection for the patient disease treatment and nutrition.^[31]^ The nursing staff actively explores the cooperation and attention to individualized management, and cooperates with other medical personnel to achieve good medical outcomes, including the improvement of patients’ quality of life, satisfaction, and treatment compliance, in line with the general goal of the current health care reform in China.^[32]^ Although intensive gastrointestinal functional interventions play an important role in improving the quality of life of gastric cancer chemotherapy patients, there are limitations to his role, and the involvement of professionals from psychological and rehabilitation disciplines may be needed to further improve the quality of life of this group of patients.^[33]^ Some domestic scholars have intervened in chemotherapy patients with gastrointestinal tumors through multidisciplinary rehabilitation teams, and the results showed that multidisciplinary rehabilitation teams can improve the quality of life and psychological health of chemotherapy patients with gastrointestinal tumors and have a positive effect on the physical and psychological recovery of patients.^[34]^ Future studies are expected to integrate individualized cluster gastrointestinal function interventions into multidisciplinary teams to further improve the quality of life of patients undergoing chemotherapy for gastric cancer.

After our study care, HR, MAP, NE, and E2 in the observation group were lower than those in the control group, indicating that accelerated gastrointestinal rehabilitation surgical cluster care combined with accelerated rehabilitation surgical cluster care can effectively improve patients’ stress response. Analysis of the reason: surgery combined with gastric cancer leads to an enhanced stress response of the body, with increased secretion of catecholamines and enhanced activity of the renin-angiotensin-aldosterone system, causing an increase in HR, MAP, and other stress-related indicators.^[35]^ Gastrointestinal accelerated rehabilitation surgical cluster care combined with accelerated rehabilitation surgical cluster care can psychologically reduce patients’ anxiety, improve their coping ability, meet their needs from psychological, physical and social aspects, and motivate them to handle the stress of surgical trauma in a good psychological state.^[36]^ Gastrointestinal accelerated rehabilitation surgical cluster care is a model that has been widely used in clinical practice in recent years, and clinical experience shows that it focuses on scientific treatment and appropriate, systematic and comfortable care services for patients, and on the stabilization of their physical and mental state.^[37]^ Early postoperative care encourages patients to perform appropriate exercise early, thus accelerating their internal metabolism, which not only helps to improve patients’ neuromodulatory functions, but also reduces their gastrointestinal tract functional excitability, which is conducive to the recovery of gastrointestinal functions, thus reducing the discomfort of the stomach caused by surgery; postoperatively, patients are instructed to chew gum for simulated feeding training, which helps to promote their liver, pancreas, bile, stomach and other organ activities It can accelerate the peristalsis of the gastrointestinal tract, increase the secretion of gastric juice and gastrin, and help improve gastric function. Abdominal massage can promote blood circulation, which can also increase the peristalsis of the gastrointestinal tract, so that the gas in the body can be expelled as soon as possible and improve the function of the gastrointestinal tract. Postoperative foot bath can stimulate the foot acupuncture points through warm water and medicine to improve the blood circulation of the whole body, which can help the normal operation of all functions of the body. Changing the position regularly after surgery can reduce the feeling of cardiopulmonary compression, while deep breathing training can help the discharge of CO2 from the body and avoid the rise of partial pressure of carbon dioxide in arterial blood. The contraction exercise can improve intestinal motility, and the contraction exercise of the anal levator muscle can also promote perineal blood circulation. Pre- and post-operative dietary health education significantly improved patients’ dietary knowledge and awareness, and comprehensive postoperative dietary guidance and supervision ensured a scientific and rational diet.

In our study, the pain scores of patients in both groups at different time points were significantly improved and the observation group was significantly less than the control group, indicating that accelerated rehabilitation surgical cluster care of gastrointestinal tract combined with accelerated rehabilitation surgical cluster care can effectively improve the pain of patients with surgical combined gastric cancer. Postoperative non-incisional pain is caused by a combination of factors, and many studies have shown that the main causes of postoperative pain are: residual CO2 in the abdominal cavity leading to pain in the back of the shoulder, quarter ribs, lower abdomen and lumbar region. The intraoperative flat position causes elevation of the diaphragm, and CO2 in the body stimulates the peritoneum and diaphragm, which aggravates subdiaphragmatic distension and intercostal pain. Non-incisional pain is caused by stimulation of the phrenic nerve pull, subcutaneous bruising at the puncture site or irritation of the hematoma.^[38]^ Intraoperatively, due to the surgical requirements, a constant lying down state is required, which tends to lead to muscle tonicity in the lumbar region, thus increasing patient discomfort. The integration of modern Chinese and Western medicine and humanistic care nursing concepts and their application to postoperative patient care interventions to predictably and rapidly and effectively prevent and alleviate postoperative non-incisional pain is a common topic faced in clinical nursing, and is also a requirement and connotation of the specific implementation of quality nursing services.

In conclusion, accelerated gastrointestinal rehabilitation surgical cluster care can effectively improve the gastrointestinal function of gastric cancer patients, improve patients’ emotional response and stress response, and has certain reference value for the care of gastric cancer patients.

## Author contributions

**Conceptualization:** Xiao-Ning Wei, Wen-Yan Cai.

**Data curation:** Xiao-Ning Wei, Fei-Ge Zeng, Kai-Ling Wu, Wen-Yan Cai.

**Formal analysis:** Xiao-Ning Wei.

**Funding acquisition:** Fei-Ge Zeng, Kai-Ling Wu.

**Investigation:** Xiao-Ning Wei, Wen-Yan Cai.

**Methodology:** Xiao-Ning Wei, Fei-Ge Zeng, Kai-Ling Wu.

**Project administration:** Wen-Yan Cai.

**Resources:** Xiao-Ning Wei, Fei-Ge Zeng, Kai-Ling Wu.

**Software:** Fei-Ge Zeng.

**Supervision:** Xiao-Ning Wei, Kai-Ling Wu.

**Validation:** Fei-Ge Zeng, Kai-Ling Wu, Wen-Yan Cai.

**Visualization:** Xiao-Ning Wei, Fei-Ge Zeng.

**Writing – original draft:** Xiao-Ning Wei.

**Writing – review & editing:** Wen-Yan Cai.
